# How to successfully select and implement electronic health records (EHR) in small ambulatory practice settings

**DOI:** 10.1186/1472-6947-9-15

**Published:** 2009-02-23

**Authors:** Nancy M Lorenzi, Angelina Kouroubali, Don E Detmer, Meryl Bloomrosen

**Affiliations:** 1 Vanderbilt University Medical Center, The Informatics Center, Nashville, TN, USA; 2 Foundation for Research & Technology-Hellas, Institute of Computer Science, Biomedical Informatics Laboratory, Crete, Greece; 3 American Medical Informatics Association (AMIA), Bethesda, Maryland, USA

## Abstract

**Background:**

Adoption of EHRs by U.S. ambulatory practices has been slow despite the perceived benefits of their use. Most evaluations of EHR implementations in the literature apply to large practice settings. While there are similarities relating to EHR implementation in large and small practice settings, the authors argue that scale is an important differentiator. Focusing on small ambulatory practices, this paper outlines the benefits and barriers to EHR use in this setting, and provides a "field guide" for these practices to facilitate successful EHR implementation.

**Discussion:**

The benefits of EHRs in ambulatory practices include improved patient care and office efficiency, and potential financial benefits. Barriers to EHRs include costs; lack of standardization of EHR products and the design of vendor systems for large practice environments; resistance to change; initial difficulty of system use leading to productivity reduction; and perceived accrual of benefits to society and payers rather than providers. The authors stress the need for developing a flexible change management strategy when introducing EHRs that is relevant to the small practice environment; the strategy should acknowledge the importance of relationship management and the role of individual staff members in helping the entire staff to manage change. Practice staff must create an actionable vision outlining realistic goals for the implementation, and all staff must buy into the project. The authors detail the process of implementing EHRs through several stages: decision, selection, pre-implementation, implementation, and post-implementation. They stress the importance of identifying a champion to serve as an advocate of the value of EHRs and provide direction and encouragement for the project. Other key activities include assessing and redesigning workflow; understanding financial issues; conducting training that is well-timed and meets the needs of practice staff; and evaluating the implementation process.

**Summary:**

The EHR implementation experience depends on a variety of factors including the technology, training, leadership, the change management process, and the individual character of each ambulatory practice environment. Sound processes must support both technical and personnel-related organizational components. Additional research is needed to further refine recommendations for the small physician practice and the nuances of specific medical specialties.

## Background

Physicians in ambulatory practices are under increasing pressure to use computer-based systems to support the clinical side of their practices. However, the rate of use of information systems for clinical care in small physician practices in the U.S. in 2006 was estimated to be within the range of 14% to 25% [1,2]. Unfortunately this pattern continues in 2008. During 2007–2008, researchers conducted a national survey of 2,758 physicians to determine the proportion of physicians who were using such records in an office setting. Four percent of physicians reported having an extensive electronic records system that was fully functional; 13% reported having a basic system in place [3]. Although not a focus of this paper, it may be useful to consider efforts underway in Europe and elsewhere to explore the implementation of electronic health records (EHRs) [4]. A 2007 Commonwealth Fund report recommended that a one percent assessment on insurance premiums and Medicare outlays should be used to finance the acceleration of provider adoption of health information technology (HIT) that encompassed decision support capacity and enabled the sharing of patient health information across sites of care [5]. The California HealthCare Foundation reported that small or solo practices or community-based clinics are far less likely to implement EHRs and electronic prescribing than physicians working in large practices [6].

This paper has two main purposes. First, it briefly presents an overview of the perceived benefits and barriers of adopting EHRs within smaller ambulatory practices in the United States, especially practices of five physicians or less. The authors build on their personal experiences of many years with academic physician practices and small ambulatory physician practices, as well as research and observation on adoption, including one author's PhD research.

The second purpose of this paper is to provide a basic guide for facilitating successful EHR implementation in smaller ambulatory practice settings for physicians and those supporting the practices. While "one size does not fit all," the authors propose a "field guide" for physician practices to illustrate some of the questions and issues that practices must address for their efforts to be successful. The authors believe that this guide is necessary to support small physician practices. The national survey mentioned above indicated that of the 83% of respondents who did not have electronic health records, 16% said that their practice had purchased but not implemented a system. An additional 26% of respondents indicated that their practice intended to purchase an EHR system in the next two years [3].

Unfortunately, most evaluations of EHR implementations in the literature are reflective of larger practice settings. Many informed observers agree that while there are similarities relating to implementation in large and small care settings, scale is both real and important. Additional and more focused research to clarify the needs of small practice settings is needed and the authors hope this paper will serve as a stimulus for such work. A step in this direction includes the June 2008 announcement that the Centers for Medicare and Medicaid Services (CMS) is implementing a five-year demonstration project that is designed to encourage small- to medium-sized (20 or fewer) primary care physician practices to use electronic health records to improve the quality of patient care. The project includes an eight year evaluation [7,8].

### Scope of discussions

This paper does not address issues relevant to the growing body of experience and literature about personal health records (PHRs) nor does it analyze implementations across countries. However, the authors recognize the significance of these issues and believe that questions relating to PHRs as well as global EHR implementation approaches warrant further discussion. The paper does not address policy implications of EHR implementations nor do we consider issues (such as barriers and benefits) related to connecting practice-based records to external information systems and records. Furthermore, the paper does not address specific EHR models for specialties within ambulatory practices and how the implementation of EHRs may need to fit within those parameters. Again, the authors recognize the need for additional discussion and research in these areas.

### Multiple visions for the EHR

Visionaries have predicted that widespread availability of EHRs in ambulatory care settings can improve the quality of care, improve communications with patients, reduce transcription costs, provide clinicians with easier cross-coverage, and support decision-making by clinicians and patients [9-13]. There are multiple definitions of an EHR. Several examples include:

• The U.S. Department of Health and Human Services (DHHS) defined EHR as "...a digital collection of a patient's medical history, including diagnosed conditions, prescribed medications, vital signs, immunizations, lab results, and personal stats like age and weight [14]."

• In June 2008, the U.S. Office of the National Coordinator for HIT (ONC) released a report proposing definitions for key health information technology terms including the electronic health record, electronic medical record and personal health record [15]. The Agency for Healthcare Research and Quality (AHRQ) stated that an electronic medical record (EMR) comprises the set of databases (or repositories) that contains the health information for patients in a given institution or organization. Aggregated EMR information derives from varied clinical service delivery processes, including laboratory data, pharmacy data, patient registration data, radiology data, surgical procedures, clinic and inpatient notes, preventive care delivery, emergency department visits, and billing information. An EHR extends the concept of an EMR to include cross-institutional data sharing. The EHR is patient-focused, spanning episodes of care rather than just one encounter [16].

• The Healthcare Financial Management Association (HFMA) outlines an electronic health record by the functions that it includes: (1) Order entry/order management – electronic management of tests, consults, and medication ordering. (2) Results management – access for physicians to all patient information for care provided by a hospital or health system. (3) Electronic health information/data capture – computerized repository storing patient health records. (4) Administrative processes – interoperable systems for billing, scheduling, resource management, and other administrative tasks. (5) Electronic connectivity – effective electronic exchange of patient data by the healthcare team and other providers. (6) Clinical decision support – computer-assisted diagnostic and disease management tools support enhanced clinical performance. (7) Health outcomes reporting – automatic extraction of information on quality indicators facilitates reporting. (8) Patient access – remote access for patients to their records [17].

• The Health Resources and Services Administration's (HRSA) Office of Health Information Technology has recently developed an adoption toolbox which is a compilation of planning, implementation, and evaluation resources to help community health centers and other safety net providers implement health IT applications in their facilities [18].

• The Healthcare Information and Management Systems Society (HIMSS) defined EHR as "...a longitudinal electronic record of patient health information produced by encounters in one or more care settings. Included in this information are patient demographics, progress notes, problems, medications, vital signs, past medical history, immunizations, laboratory data, and radiology reports. The EHR automates and streamlines the clinician's workflow. The EHR has the ability to independently generate a complete record of a clinical patient encounter, as well as supporting other care-related activities such as decision support, quality management, and clinical reporting [19]."

The definitions for and expectations of EHRs have expanded in scope as information systems technology and the discipline of informatics have evolved and matured. Currently, the concept of EHRs incorporates a full range of functionality and interconnectivity and this range is a challenge for small practices.

Before embarking on an EHR implementation project, it is important for the practice to have realistic expectations. It is also critical to be familiar with generally recognized barriers and benefits. Thus, before providing guidelines for the implementation itself, we offer a synopsis of what is known about benefits and barriers with special attention to those issues most relevant to smaller practices. This section is not intended to be a comprehensive review of benefits and barriers but is provided to illustrate the key issues that small practices are likely to encounter.

## Discussion

### Benefits of EHR use in ambulatory settings

Benefits of an electronic health record in ambulatory practices fall within three main categories: improved patient care through more efficient access to accurate records; improved office efficiency; and potential financial benefit.

#### Improved patient care

An electronic health record has the potential to strengthen the quality of care and the relationship between clinicians and patients through ready access to accurate and up-to-date patient information from office or remote locations. Baron et al. noted that, after implementing an electronic health record system, the Greenhouse Internists (4-internist, community-based practice of general internal medicine located in Pennsylvania) "...communicate more quickly and clearly with patients on the telephone and by letter, transmit important clinical information more efficiently to specialists, and spend less time paging through charts....". Greenhouse Internists has operated in Philadelphia since 1989 and serves an economically and ethnically diverse urban and suburban population. Baron et al. reported that the Greenhouse Internists' patients were impressed upon seeing their prescriptions appear electronically [20]. It is also reported that these EHRs provide the opportunity to access national databases, such as the National Cholesterol Education Program Risk Calculator [21] for patient use between visits.

#### Improved office efficiency

The patient's chart can be located in multiple places, e.g., the physician's private office, waiting to be filed, with the nurse, or filed. An EHR saves staff time otherwise used searching for charts, entering charges manually, etc. Depending on the size of the practice, this "found time" can be devoted to value-added activities or eliminated, thereby reducing overtime charges. Through the use of EHRs, productivity increases because of improved office efficiency. If a half hour of paperwork is eliminated, that could mean two more patients seen daily or 30 more minutes a provider could spend at home with family members [22].

Miller et al. interviewed providers with EHR systems [23]. They reported that providers worked longer hours for an average of four months during initial EHR implementation, mostly because of inefficiencies while on the "steep" part of the software learning curve and due to the one-time requirement of entering all clinical data during each patient's initial visit after implementation. The study found that quality of life improved for many providers after the initial implementation period. Three practices benefited from seeing the same number of patients in less time, taking the gain as more personal time, rather than as an opportunity to see more patients. Providers in most practices particularly liked accessing records from home, which enabled some of them to go home earlier. The providers also characterized as an improvement the ability to access records immediately while on call.

#### Potential financial benefits

Baron et al. reported that the Greenhouse Internists Group had a total budget for technology support of $10,000 per year before implementation of the EHR to support and maintain their practice management system and their limited network.

The post-implementation annual budget was approximately $40,000 for hardware and software vendors and a substantially increased usage of a local computer support vendor. Year-one hardware and software acquisition costs depreciated at $24,000 annually over a 5-year time period. On the "gain" side, the group eliminated one staff position ($20,000) and $45,000 in annual transcription costs. The group expects to see more patients during the same amount of time or to transfer physician work to other members of the office staff more reliably and safely because the system provides clear, timely, and legible documentation to support expanded clinical team activities. Within one year of implementation, the group expected to free their file room space and make it clinically productive [20]. Some anecdotal reports suggest that billings increase a few percentage points after implementation [24].

### Barriers to EHR use in ambulatory settings

Several obstacles have been cited as explanations why EHRs have not achieved more prevalent usage in physicians' offices. These obstacles include:

• EHR products are expensive and require a major investment;

• EHR applications are not standardized;

• EHRs are more difficult to use than paper-based records;

• EHR implementation reduces practice productivity and disturbs workflow (at least initially);

• EHR benefits accrue to others (such as society and payers) not to providers.

A study by Gans et al. confirmed that the top barriers that physicians list are the cost of the systems, clinicians' concerns about technically supporting a system, and clinicians' ability to use the new system [25]. Baron et al., in describing the lessons learned by the Greenhouse Internists group in implementing the EHR system, stated, "It is naïve to assume that small practices will move to EHRs without a variety of supports, one of which is certainly financing.... Enhanced reimbursement models will be needed for wider adoption [20]."

In practices with EHRs implemented, Gans et al. found that the main impediments experienced included "...'people barriers' – lack of support for the system from physicians, non-physician providers, and other clinical staff." Physicians who implemented EHRs and those that have not cite the lack of capital resources and concerns about loss of productivity as major issues. Overall, the study concluded that the transition from computer-based administrative information systems to fully-implemented EHRs is a major undertaking that creates dislocation among the clinical staff and is more complicated, more difficult, and more expensive than most practices expected [25].

Simon et al. conducted a survey of a stratified random sample of 1,829 office practices in Massachusetts in 2005. The survey measured use of health information technology, plans for EHR adoption and barriers to adoption as perceived by the practices. Simon found that in Massachusetts, less than 1 in 5 practices use EHRs and that even among adopters there was considerable variation in use by functionality and across practices. Many practices do not use EHR functionalities needed to improve healthcare quality and patient safety. Simon also found that among practices that do not have EHRs, more than half lack plans to adopt them and that lack of funding is a key barrier to EHR adoption in ambulatory care practices [26].

The authors believe that some of the items listed above are true barriers and others are "pseudo" barriers caused more by general resistance to information systems for the forced changes they impose on long-established practice habits rather than the systems themselves. Physician resistance to information systems has been extensively discussed in the literature as an important barrier to EHR adoption [27-29]. However, based on implementation experiences witnessed and studied by the authors, the authors believe that physicians are now more willing to adopt new technologies when the applications are user-friendly and fit within their daily workflows. Since healthcare providers are willing to use technologies that meet their needs, then the processes of selecting and buying, planning for implementation, and carrying out system implementation must be considered, especially for small practice settings, since to date, most vendor systems have largely been designed for larger practice environments.

### Introduction to change as a key factor in EHR implementation

Practical experience has shown that change is an ongoing process of anticipated, emergent and opportunity-based events that have a fluid and unpredictable nature. People who work together closely on a daily basis are the individuals who initiate change in smaller ambulatory practice environments. Resistance in this type of environment is often temporary, as there is a tendency for smaller organizations to seek a steady equilibrium [30]. Nevertheless, change management cannot address the external financial and policy barriers mentioned previously. Additionally, the complex issues related to privacy, security and confidentially are beyond the scope of this paper. However, once a decision is made to implement EHRs, managing change is invaluable to the process.

Lessons of change management from larger institutions cannot be easily or directly applied to unique ambulatory healthcare practice settings. The cultures that comprise healthcare settings (e.g., physician offices, hospitals, surgery suites, etc.) add to the complexity of change efforts. The nature and the organizational variation of physician practices require an approach to change that is flexible. The smaller ambulatory practice change environment emphasizes individual enthusiasm, commitment, and personal ability of individuals to share information and to cooperate. Individuals within such practices who have adequate technical knowledge and skills are in a better position to assist and support the entire office during an EHR implementation than are such individuals in much larger environments. Small ambulatory practices place a greater emphasis on managing relationships at the core of the new behavior that the practice wishes to instill [31]. This view of change as internally generated is relevant in ambulatory physician offices. While most physician practice leaders can find the financial resources to support an EHR, the requirements and decisions for the appropriate EHR system come directly from the entire practice.

To be successful, physician practice groups need to place attention on the practical aspects of EHR implementation. The technology must be easily installed and maintained, supported locally, easily understood and controlled by local users, be flexible and adaptable to the needs of different healthcare personnel, and be organizationally simple, while requiring low investment at each site [32]. Overall, an important first step in achieving change is for those involved to realize that change is possible. Unless there is local "ownership" of the project and the process of change, local commitment to solve the inevitable problems that arise, local expertise to train and motivate the people in the front line of action, and local ability to assemble appropriate resources and support, EHR implementation is unlikely to succeed. Further, it is crucial in small practice settings not to overlook the critical roles played by non-physician members. Everyone in the practice needs to be involved. The implementation champion in the practice setting need not be a physician as long as there is agreement that the change does need to come and be led by someone who is highly respected. A study commissioned by Canada Health Infoway provides a comparative analysis of automation in general practice in 10 countries. The study notes that if a physician is not a champion, a practice administrator can play that role [33].

In the authors' experience, impediments to adoption include the difficulty of understanding the information needs, the uncertain cost implications of implementing a system, and the intense effort required to identify and implement a system. In order to overcome implementation obstacles, it is important to be clear on what the EHR will bring into a practice before implementation begins.

### Steps toward successful ambulatory EHR implementation

A decision to implement an EHR requires a considerable amount of time. If a practice does not have the time to understand what an electronic health record can do, to investigate and decide on what system to buy, to implement the EHR, to train everyone, and to continuously monitor the system, it is better to wait until the time is available to invest. However, if the practice is ready to implement an EHR, there are basic practical implementation steps to ensure that the probability of success will increase dramatically. Readiness is a key first factor along with the eagerness of personnel, availability of a champion, perceived usefulness of the EHR and teamwork. One of the most important lessons that people working in this field have discovered is that people-based skills (cooperation, leadership, creative thinking) are as important as the technology itself [34].

As Baron's Greenhouse Internists experience indicated, planning must address the initial effect of EHR implementation on the clinical practice and the corresponding transient reduction in practice efficiency. Baron et al. stated, "Perhaps the most important asset we could have used to ease the pain of implementation was more clinical capacity. A decline in productivity after implementation...seems inevitable, and if a practice is already straining to meet patient demand, an absence of reserve magnifies the stress of implementation [20]." In the following section we describe essential steps in the process of EHR implementation: creating a vision, phases of implementation, key role of the clinical champion, and workflow redesign.

The first step begins not by thinking about an EHR for the practice because other practices have one, but by thinking about how members of the practice would like their practice to operate in the future. With this focus, an EHR is about implementing the *vision*, rather than technology [35].

### Creating a vision for CHANGE

The keys to successful creation of a vision are having the leadership to begin this process with a "can do" attitude and having or gaining knowledge and understanding of the needs of all the physicians and staff, the patients, the health systems with which the practice is affiliated, and other questions relevant to the practice.

To create an actionable vision, all members of the office staff must answer questions, such as: (1) How would you like to practice medicine within the next 5 years? (2) What goals does the vision incorporate? (For example, improved/more rapid clinical decision-making; better quality of patient care; rapid and convenient access to patient information; and more rapid response to telephone calls and/or decreased number of telephone calls to pharmacies, etc. (3) What type of tools/technology does each person envision using?

The vision provides the foundation that allows creation of EHR capabilities statements. This step translates the vision into workable and understandable action-oriented goals. As an example of the concepts described above, a *vision statement *might be:

#### Example of a Vision Statement

*Our office practice will have electronically integrated information available to effectively support the clinical care of our patients*.

Several of the many supporting capability statements might be:

◦ *The system will be capable of making multiple uses of the information that has been entered*.

◦ *Our system will support access in a secure manner from remote locations, other than just office locations*.

◦ *Our system will assist with evidence-based decisions by providing access to information from the evidence-based literature*.

These examples demonstrate how "capability" statements begin to help the clinical practice focus on what is most important for their office or clinic. The following is an outcome example of a physician practice first creating a vision and then investigating options.

#### A Vision Experience Example

*Non-affiliated private practice physicians in the community provide about 40% of the total ambulatory care volume for an inner city area with a population of about 240,000 people. The size of practices within this Community Health Group varied, from 3–20 employees. Even in the smaller practices with only 2 doctors, both had to be aware of the benefits of the EHR system in order to use it. Often, one had to act as the champion to promote the EHR implementation. The reasons of using an EHR are not necessarily obvious to physicians. It is, therefore, important to be clear on what the EHR will bring into the practice. In small practices, even though one doctor wanted to adopt the EHR, the others often did not, and the implementation did not proceed. When physicians were shown the capabilities of the new system, their eyes lit up and were anxious to get the system as soon as possible. In an inner city environment, many patients have low literacy levels or speak Spanish, making communication difficult. Others would lose the exam results printed on paper, or forget them. To overcome these difficulties, physicians were keen to access their patients' hospital records and especially the results of laboratory exams electronically to ensure continuity and timeliness of care. A doctor illustrates the need: "I had never used a computer before. However, I learned how to access my patients' records at the hospital from my practice. It was very useful for me. Having the electronic number of the patient, I could view their medical exams, history, and diagnosis." Another doctor concludes: "Accessing the hospital EHR saved us time, reduced errors, and improved efficiency of care for our patients *[36]."

### Phases of EHR implementation

EHR implementation can be characterized by several phases: Decision, Selection, Pre-Implementation, Implementation, and Post-Implementation. Each phase has its key issues to address. As practices vary in size, culture, capacity, knowledge of information systems, and staffing, the following is provided as a "field guide" toward successful implementation of an EHR in an ambulatory practice. Tailoring the approach to the individual practice is critical.

#### Decision phase

The Decision Phase focuses on identifying champions, gaining "buy-in," collecting information, assessing workflows, understanding financial issues, and analyzing benefits.

##### Identifying champions

A champion is an absolute necessity for a successful implementation. The optimal approach is to identify one of the most *clinically-respected *providers who has technology knowledge and who is committed to an EHR to fulfill this champion role. A practice champion provides direction and inspires, encourages, promotes and creates trust in the process, and in the future. In return, everyone in the practice needs to trust, respect, and communicate effectively with the champion. Champions must provide a combination of control and flexibility to create the highest likelihood of implementation success. It is important to re-emphasize the overall value of a champion to successful adoption [37,38]. The following is a brief experience of a physician champion.

###### A Sample Physician Champion Experience

*One of the key doctors in a medium size clinic describes his role as follows: "I do double work promoting teamwork, establishing EHR activities, however, when I see my efforts paying off, I do not regret the time I put in." A major challenge was based in cultural issues. Promoting the EHR was greatly facilitated through a physician that was very active in the practice and community. He was well respected among his peers and acted as a liaison between the practices, local community, and the hospital. His intimate knowledge of the organization of community clinics, the staff, the needs, and requirements helped the implementation team better understand the community. The physician was a leader encouraging clinical staff to use the EHR. He shared his vision of information exchange among practices, better communication, and opportunities for practice improvement. Even doctors that were not familiar with information system capabilities started to share the same vision*.

##### Gaining "buy-in"

A fast track to project failure involves lack of planning for the emotional side of change. Lorenzi et al. noted that, "the technically best system may be woefully inadequate if its implementation is resisted by people who have low psychological ownership in that system. On the other hand, people with high ownership can make a technically mediocre system function fairly well [38]."

To gain buy-in within an office practice, communication and involvement are crucial components. Early and effective communication to all members of the practice, starting at the first consideration of an EHR, is a key strategy for staff involvement. Leaders must encourage all members of the practice to provide input into the process, to set expectations, and to anticipate and report potential strengths and weakness of an EHR implementation within the practice. Early participation prepares the staff for the extensive involvement needed during the implementation period. Involving people from the very beginning helps them to feel part of the process and the solution.

##### Collecting information

Champions and clinical staff must identify what data needs to be included in the EHR system and must identify the definitive source of each data item. Possible information includes: (1) All patient data including records of telephone messages and scanned versions of outsiders' correspondence. (2) Radiological reports and possibly digital images from outside imaging centers. (3) Electronic abstract, including discharge summaries and laboratory data, from one or more hospitals.

One of the first steps in deciding to adopt an information system is to gather accurate performance data for the existing system(s) – whether electronic or paper. A commonly encountered form of resistance to new system implementation is the complaint that the new system compares unfavorably to the old system. While presenting factual data does not counteract emotional reactions, it is important to address unfounded allegations or rumors about the new system.

At the information collection stage, it helps for the EHR champion to gain additional expertise about the subject by taking some of the short courses available (e.g., the National Library of Medicine's short course in Biomedical Informatics offered twice a year at Woods Hole laboratories http://courses.mbl.edu/mi/, or courses offered as part of the American Medical Informatics Association's (AMIA) 10 × 10 initiative http://www.amia.org/10x10) or by visiting a few places that are known to be doing a good job with EHRs in their practices.

##### Assessing workflows

An American Academy of Family Physicians (AAFP) survey found that 54.2% of 5,000 respondents worried about the possibility of a slower workflow and lower productivity when an EHR is installed [39]. Studies document that an EHR that does not integrate smoothly into clinicians' workflows and that does not allow for variations in style can adversely affect productivity and financial return on investment [40].

To address these concerns it is important to understand and to document the multiple workflows within the current office practice, e.g., how appointments are scheduled, what occurs during an actual patient visit, what are the workflows after the patient visit, how the office practice handles unscheduled patient visits, questions, etc. Assessing workflows is a pre-requisite for determining possible impacts of the EHR on office practices, and for the important process of workflow redesign prior to implementation of the EHR. Workflow redesigns that are completed and tested before a new system is introduced can help prevent "blame" for problems directed at the new information system and/or champion/leaders following system "go live."

Another important workflow consideration is how the office or clinic will "survive" during unanticipated system downtime. If the only form of patient records is fully electronic charts, and the system is "down," will patients be sent home or to another facility to receive care? Are there adequate "back ups" and redundant servers locally so that the office can continue to operate based on local resources? Failure to adequately plan for downtime can cause catastrophic effects on clinical practices during actual downtime events.

##### Understanding financial issues

Many physicians express concern about the lack of financial support for startup costs, including costs for setting up the EHR, the technology, and the training [23]. Additional costs to the practice may accrue from decreased patient care efficiencies immediately post-implementation, as noted by Baron et al [20]. It is important for the physician practice to understand the total scope of the costs associated with the EHR that are beyond the initial purchase price. The practice needs to analyze the costs mentioned and determine the "return on investment," or at least the price they are willing to pay for specified improvements related to the purchase and installation of an EHR.

##### Analyzing benefits

An analysis of the benefits of an electronic health record system involves both the financial as well as non-financial benefits that can accrue to a practice once the EHR is fully functioning. A number of the benefits listed will come from the practice vision statement. During this stage, the metrics and methods are created for monitoring the benefits that are of interest to the practice. After the practice makes the decision to install and support an electronic health record system, the next step is to investigate options and select an EHR to implement.

#### Selection phase

Deciding whether to move to an electronic health record system and which system to choose can be very strenuous. This article does not focus on EHR systems or system selection. Nevertheless, a few points of advice based on the authors' experience apply.

• Few if any ambulatory practices can develop their own EHR system, therefore, a commercial vendor is often the likely source of the product selected. An alternative is to investigate a shared EHR system from the hospital or healthcare system affiliated with the ambulatory practice.

• Open source options such as versions of the VA Veterans Health Information Systems and Technology Architecture (VISTA) [41-43] system are also now gaining momentum as are Internet-accessible approaches [44].

• Many vendors are stronger on sales than on support, therefore it is critical to find a vendor with a reasonably large, satisfied customer base that includes practices similar to one's own practice.

• Visiting practices that have installed the system of interest is essential to learn about the "hidden costs" and the problems likely to be encountered and the responsiveness of the vendor to problems, and to obtain advice on how to overcome common problems.

• If visiting is not possible, talk with more than one practice using the potential system.

• Ask the potential vendor to provide access to a demonstration system for all practice members to "test-drive."

• Ask all staff for their assessment of the strengths and weakness of the system as they perceive that the system would apply to the practice.

• The wording of the contract to purchase and support the system can make or break EHR implementation success. Base payments on achieving functional milestones determined by the practice, not by the vendor.

• The Internet provides a valuable source of information regarding specific EHR system products, capabilities, and the selection process. For example:

• Edsall et al. surveyed 408 family physicians with EHRs and published the results of their survey [45].

• The state of West Virginia through its e-Health initiative published information about purchasing an EHR for solo and small group practices [46].

• A basic primer for EHR system review and selection [47].

• EHR selector services (for a fee) that direct physician practices to the EHR that might be best for their practice [48].

Whatever the process, it is important to spend the time required to understand both the practice needs and the capabilities of the EHR systems on the market that can adequately meet those needs.

#### Pre-implementation phase

A decision is made to move forward with implementing an EHR. The steps within this phase include: communicating and involving people – staff and patients; redesigning workflows; establishing a project plan; getting help; timely training; and having fun.

##### Communicating and involving people

The crucial elements for a practice preparing to implement an EHR are people, planning, leadership, and implementation processes. The key to success is the involvement of people – those connected to the practice and patients. Participation in the assessment and implementation of the EHR will ensure that individuals' information needs are considered and addressed. In turn, the people will have a greater investment in the success of the system. To gain the confidence of everyone, communication is a major cornerstone. Everyone in the practice must know about the EHR project plus the goals and the plans for implementation. These actions initiate the "buy-in" process and prepare the staff to respond to any patient questions.

##### Redesigning workflow

A well-run physician practice office is a complex operation with well-defined workflows. Principles that influence the redesign of workflows include simplicity, accessibility for patients, safety, comprehensiveness of documentation, and delegation. The Greenhouse Internists assumed that the physician, being the most highly skilled as well as the most expensive person in the practice, should only do what no one other than a physician could do. To move forward, the group redesigned every office system. They reviewed and adjusted their workflows during the EHR implementation [20].

##### Establishing a project plan

There are many views about project management. The following issues are useful to determine the success and failure of health informatics projects of any size.

• *Clarity of responsibility*. One person needs to be designated as the leader or coordinator of the effort. This person is most likely the champion. Clearly defined lines of communication and responsibility promote progress and effective reporting.

• *Setting objectives*. The first step in managing the project is the setting of realistic objectives and timelines. All significant parties involved need to commit emotionally and display ownership of project objectives. Obtaining early project ownership among staff requires a participative approach. The objectives include specific, realistic definitions of project success. Until this stage is completed, no further work should proceed.

• *Action planning*. Generally a project plan defines its action steps in terms of major steps with specific start and end dates for each step. The planning process then moves to the next lower level of detail. As successively lower levels of detail are reached, project leaders need to seek input from the practice staff. This is critical to obtain both their valuable input and their psychological commitment.

• *Tight control and feedback procedures*. An organized system must be designed and put into place to obtain timely feedback on the status of each portion of the project. It is critical to obtain the earliest possible warning of any deviations from schedule or budget – positive or negative.

• *Ongoing problem solving*. Unforeseen problems arise in virtually every project, although quality planning does help to reduce them. As problems do arise, they must be dealt with by a problem-solving approach – not a finger-pointing one. Finger pointing and "blaming" generally lead to negativism, defensiveness, and the temptation to seek revenge – all fatal to project success.

• *Project completion*. As the project approaches completion, an evaluation process should begin to measure the success of the project against the original success criteria. In fact, evaluation should be incorporated into an ongoing monitoring and improvement process within the practice.

##### Getting help

A contingency plan for obtaining help and support needs to be included in the original plan. Do not save this until a serious problem suddenly looms. Decisions about who will handle initial problems as well as how to escalate the process – both inside and outside of the practice – need to be considered and defined.

##### Conducting training

There is increasing recognition that training, effective change support and stakeholder education are key to a successful transition to an EHR [49-51]. Quality training can help significantly in reducing anxieties about using a new system. The availability of technical and training support during the initial implementation is essential [52]. Timing of training is critical. Training that is either too early or too late will waste resources and raise frustrations. The technology introduces the required tools to transform daily work, and training introduces the requisite skills to do it. The nature of technology has both a facilitating and a hindering effect. The design of the technology incorporates assumptions about its use that are not always congruent with the goals of the ambulatory practice members. Training must be brief, high-quality, closely timed to the point of need, and specifically directed to the practice's staffing and needs. Training needs to include a "practice" version of the system. Good training does more than build skills; it continues the communication and involvement opportunities. There are multiple audiences to be considered when planning training associated with EHR implementation and tailoring training strategies and plans to different subgroups (physicians, nurses, practice managers, receptionists, physician extenders) makes sense.

##### Having fun

Whenever possible, project change leaders must introduce elements of fun. Two fun techniques used previously are lunch-time or end-of-day training and planning sessions that provide pizza and soft drinks, and sessions that feature some form of non-threatening competition (e.g., between physicians using the system and physicians not using the system, or between nurses performing physician-related functions on the system and physicians performing nurse-related functions). This also provides an excellent opportunity to talk about some of the interesting experiences during the selection process. The message is that facing the future does not need be grim!

###### One Author's Implementation Observation

*In medium size practices of more than two doctors, the existence of teamwork is very important in incorporating the EHR in daily practice. Physicians should be working closely together as a team in order to agree on electronic information sharing through a common EHR. In practices where doctors did not communicate well, had other priorities, or did not want to share electronic data, resistance towards EHR implementation was very common. On the other hand, in practices where sharing of information, communication, and collaboration in patient care were well established, the use of EHR as an instrument for continuity of care was easily understood. As the practice of medicine is inherently autonomous, teamwork is not automatically established but gradually developed. The role of a champion to promote teamwork and information sharing is imperative for the success of EHR implementations*.

Large healthcare institutions usually have technical support staff for supporting and maintaining systems. In contrast, there was no support staff located in community physician offices. The configuration of the system has to be robust and stable in order to avoid extensive support and maintenance. In practices where doctors also had technical computer skills, implementation of the EHR proceeded faster as the process was assisted from within the practice. Physicians with technical computer skills acted as champions and promoters of the EHR system. Practices with such personnel had an advantage over other practices because they relied on personal initiative and internal skills. A nurse describes her experience in the initial stages of the implementation process: "The whole task felt impossible. We had so many questions that it was impossible to call the help desk during a busy day. Thankfully, Dr. Smith would come and ask us if we had any problems many times during the day. We felt supported and at ease with the new system. We knew that we could ask him any question without having to interrupt our workflow."

#### Implementation Phase

This phase assumes that realistic expectations were developed. If physicians and other key office staff are oversold on what the new system will do, the system is doomed to be regarded as at least a partial failure. The EHR champion must help the practice set realistic expectations for the impact on initial productivity during the early system implementation stages. During the implementation of an EHR, practice productivity will initially decline, no matter how good the system and what the preparations are for its implementation.

The following concepts must be addressed during the implementation process: engaging the patient; making changes and managing change; implementing rapidly and supporting extensively; and encouraging the practice.

##### Engaging the patient

Patients, especially those who visit more frequently, know when changes occur. Informing the patients about the anticipated EHR and what it will mean for them is important. Some practices develop a one-page handout to tell more about what will happen, when, and potential inconveniences and planned benefits for the patients. Early patient communication and involvement is useful.

##### Making changes and managing the change

No EHR system can be used immediately "as delivered," nor can any EHR system totally satisfy the needs of a busy practice. Given this reality, it is important at the pre-implementation stage and during implementation to identify the practice needs to customize the selected system.

Each practice is unique in terms of its dynamics. Understanding the environment facilitates change management. Champion leaders need to identify key issues as they arise and address them as rapidly as possible. A change management strategy generally includes mechanisms for soliciting feedback at all stages of the change process. The alternative of not identifying problems and not providing feedback about problem resolution leads to misinformation within the office practice. Feedback obtained must be addressed promptly. Every issue cannot be resolved to everyone's satisfaction, but sharing information about which issues can be addressed (or not) and in what time frame is important.

##### Implementing rapidly and supporting extensively

When it is time for the actual implementation, complete the implementation as rapidly as possible and provide ample support. A primary goal is to have adequate personnel for direct support. Supplementary support in the form of written manuals, "how to" laminated cards, and online tutorials can also address the varied learning styles of individual users.

##### Encouraging the practice

Celebrating change-related milestones remains important. As noted in the studies of Lorenzi et al., throughout an implementation effort there are many people who contribute directly or indirectly [37]. The people who are the "heroes" for their efforts in the implementation process should be acknowledged and honored. Practice leaders need to reassure people about the changes that have taken place. Celebrations bring people together in a relaxed and informal setting to laugh a little and celebrate the success. It is important to stress that this is a celebration of reaching a *significant milestone on a long journey*, not an arrival at a destination.

#### Post-implementation phase

The post-implementation phase involves continuous updating, training, evaluation, and again, celebration. Typically information systems have "updates" on a routine basis. When an update occurs, system users must be informed about the changes and re-trained if required. Each change to the system has implications for the daily work of the practice. Failure to continuously educate will cause individuals or the entire practice to "fall behind," with resultant problems in system use and practice productivity.

Evaluating the process of implementing an EHR is significant. Did the implementation process occur smoothly? Did everyone in the practice participate and feel involved? Did events occur as planned? What were the strengths and weaknesses of the implementation? Evaluating the actions that occurred and the staff's reaction to them helps to shape both the practice and its future evolution. Very often what happens during an implementation is very different from what was planned. It is important to know what happened to either avoid repeating mistakes in the future or to follow a similar path to success at a later time.

Continue celebrating the new information system through sharing information and taking time to recognize and share success with the entire staff and with patients.

## Summary

Ambulatory practices are drawn toward teamwork, quality healthcare, patient information and support, and meeting patient needs. The EHR implementation experience depends on a variety of factors such as the technology, training, leadership, the change management process, and the individual character of each ambulatory practice office environment. The combination of these factors leads to differing implementation experiences.

This article presented a review of the benefits and barriers of EHRs for ambulatory clinical practices. To the extent possible we have identified studies concerning the implementation of EHRs in ambulatory settings in general and in small physician practices in particular. The goal is to provide a practical "field guide" for success based on experience with ambulatory practices of various types, sizes and locations. The key to success is to know how to enlist the right processes and resources to support the needs of individual practices during EHR implementation. Good, sound processes must support both technical and personnel-related organizational components. Both are important. Success is defined as the implementation and use of EHR technology to meet or exceed the stated vision and goals. Success often comes at the price of temporary setbacks and unanticipated frustrations. The result of successful EHR implementation is that the quality of patient care improves.

The majority of physicians and other healthcare providers readily learn, collaborate, and transform their daily work [31]. No matter how difficult the transition stage is, once the new system and workflows are in place, it is unlikely that the practice will want to revert to the old processes. Naturally, additional research is needed to further refine applicable recommendations for the small physician practice and the nuances of specific medical specialties. In spite of the dynamic nature of the industry and the increased implementation of EHRs across various settings there is a need for additional research concerning this subject in order to adequately understand and document the potential for increased efficiencies and potential benefits in smaller practices.

## Competing interests

The authors declare that they have no competing interests.

## Authors' contributions

The authors contributed equally to this manuscript. All authors read and approved the final manuscript.

## Pre-publication history

The pre-publication history for this paper can be accessed here:

http://www.biomedcentral.com/1472-6947/9/15/prepub

## References

[B1] Robert Wood Johnson Foundation, Electronic Health Records Still Not Routine Part of Medical Practice, Says New Study 2006 http://www.rwjf.org/pr/product.jsp?id=21882

[B2] Baldwin Gary News Feature: Paper Charts No More (Data from Medical Group Management Association) 2006 http://www.qualitynet.org/dcs/ContentServer?c=MQNews&pagename=Medqic%2FMQNews%2FNewsFeatureTemplate&cid=1149260508636

[B3] DesRoches CM Campbell EG Rao SR Donelan K Ferris TG Jha A Kaushal R Levy DE Rosenbaum S Shields AE Blumenthal D Electronic Health Records in Ambulatory Care – A National Survey of Physicians N Engl J Med 2008 359 1 50 60 10.1056/NEJMsa0802005 18565855

[B4] Benchmarking ICT use among General Practitioners in Europe 2008 http://www.ehealthnews.eu/content/view/1113/62/

[B5] Schoen C Guterman S Shih A Lau J Kasimow S Gauthier A Davis K Bending the Curve: Options for Achieving Savings and Improving Value in U.S. Health Spending 2007 xi http://www.commonwealthfund.org/Content/Publications/Fund-Reports/2007/Dec/Bending-the-Curve--Options-for-Achieving-Savings-and-Improving-Value-in-U-S--Health-Spending.aspx

[B6] California HealthCare Foundation The State of Health Information Technology in California: Use Among Physicians and Community Clinics 2008 http://www.chcf.org/topics/view.cfm?itemID=133640

[B7] Department of Health and Human Services Electronic Health Records Advancing 21st Century Medicine http://www.hhs.gov/news/facts/ehr21stcentury.html

[B8] Centers for Medicare and Medicaid Services Medicare Demonstrations: Details for Electronic Health Records Demonstration http://www.cms.hhs.gov/DemoProjectsEvalRpts/MD/itemdetail.asp?filterType=none&fi lterByDID=-99&sortByDID=3&sortOrder=descending&itemID=CMS1204776&intNumPerPage=10

[B9] Greenes RA Pappalardo AN Marble CW Barnett GO Design and implementation of a clinical data management system Comput Biomed Res 1969 2 5 469 85 10.1016/0010-4809(69)90012-3 11697375

[B10] Gifford S Maberry D An integrated system for computerized patient records Hosp Community Psychiatry 1979 30 8 532 5 313361 10.1176/ps.30.8.532

[B11] Neuhaus E Lyons TF Payne BC Problems of case finding and data collection in ambulatory care settings Am J Public Health 1980 70 3 282 3 7356094 10.2105/AJPH.70.3.282 PMC1619377

[B12] Walker CH Child health records and computing Br Med J (Clin Res Ed) 1982 285 6355 1671 2 6814702 10.1136/bmj.285.6355.1671 PMC1500799

[B13] Barnett GO Winickoff RN Morgan MM Zielstorff RD A computer-based monitoring system for follow-up of elevated blood pressure Med Care 1983 21 4 400 9 10.1097/00005650-198304000-00003 6341724

[B14] HHS announces initiative plans for national electronic health record system http://amcnoma.org/webpages/main/hhs_announces_initiative_plans_060905.htm

[B15] National Alliance for Health Information Technology Defining Key Health Information Technology Terms 2008 http://www.hhs.gov/healthit/documents/m20080603/10_2_hit_terms.pdf

[B16] AHRQ Electronic Medical/Health Records http://healthit.ahrq.gov/portal/server.pt?open=514&objID=5554&mode=2&holderDisplayURL=http://prodportallb.ahrq.gov:7087/publishedcontent/publish/communities/k_o/knowledge_library/key_topics/health_briefing_01232006114616/electronic_medical.html

[B17] Healthcare Financial Management Association Overcoming Barriers to Electronic Health Record Adoption: Results of survey and roundtable discussion conducted by the Healthcare Financial Management Association 2006 http://www.hfma.org/NR/rdonlyres/4FE68E23-0A47-4674-ABBA-F1A4AA1E73A9/0/ehr.pdf

[B18] HRSA Health IT Adoption Toolbox http://healthit.ahrq.gov/portal/server.pt?open=512&objID=1077&PageID=14253&mode=2&cahced=true&wtag=wtag503

[B19] HIMSS EHR Association Membership Committee HIMSS EHR Association Definitional Model and Application Process, 2006 October http://www.himssehra.org/docs/EHRVA_application.pdf

[B20] Baron RJ Fabens EL Schiffman M Wolf E Electronic Health Records: Just around the Corner? Or over the Cliff? Ann Intern Med 2005 143 3 222 6 16061920 10.7326/0003-4819-143-3-200508020-00008

[B21] National Heart, Lung and Blood Institute Risk Assessment Tool for Estimating Your 10-year Risk of Having a Heart Attack http://hp2010.nhlbihin.net/atpiii/calculator.asp

[B22] Adler KG Why It's Time to Purchase an Electronic Health Record System Fam Pract Manag 2004 11 10 43 6 15575139

[B23] Miller RH Sim I Physicians' Use of Electronic Medical Records: Barriers and Solutions Health Aff (Millwood) 2004 23 2 116 26 10.1377/hlthaff.23.2.116 15046136

[B24] Miller RH West C Brown TM Sim I Ganchoff C The Value of Electronic Health Records in Solo or Small Group Practices Health Aff (Millwood) 2005 24 5 1127 37 10.1377/hlthaff.24.5.1127 16162555

[B25] Gans D Kralewski J Hammons T Dowd B Medical Groups' Adoption of Electronic Health Records and Information Systems Health Aff (Millwood) 2005 24 5 1323 33 10.1377/hlthaff.24.5.1323 16162580

[B26] Simon SR McCarthy ML Kaushal R Jenter CA Volk LA Poon EG Yee KC Orav EJ Williams DH Bates DW Electronic health records: which practices have them, and how are clinicians using them? J Eval Clin Pract 2008 14 1 43 7 18211642 10.1111/j.1365-2753.2007.00787.x

[B27] Lorenzi NM Riley RT Managing Technological Change: Organizational Aspects of Health Informatics 2004 New York: Springer-Verlag

[B28] Edelson J Physician use of information technology in ambulatory medicine J Ambul Care Manage 1995 18 3 9 19 10143483 10.1097/00004479-199507000-00004

[B29] Teach RL Shortliffe EH An analysis of physician attitudes regarding computer- based clinical consultation systems Comput Biomed Res 1981 14 6 542 58 10.1016/0010-4809(81)90012-4 7035062

[B30] Wiener C Fagerhaugh S Social organization of medical work 1985 Chicago, University of Chicago Press

[B31] Kouroubali A Implementation of Health Care Information Systems: Key Factors and the Dynamics of Change PhD Thesis 2003 University of Cambridge, Cambridge, UK

[B32] Clegg D Appropriate technology for humans and organisations J Inform Technol 1988 3 3 133 145 10.1057/jit.1988.29

[B33] Protti D Comparison of Information Technology in General Practice in 10 Countries Healthc Q 2007 10 2 107 16 17491575

[B34] Sittig DF The Importance of Leadership in the Clinical Information System Implementation Process http://www.informatics-review.com/thoughts/leadership.html

[B35] Lorenzi NM Clinical Adoption Aspects of Electronic Health Records 2006 2 New York: Springer 378 397

[B36] Kouroubali A Starren J Barrows C Clayton PD Practical lessons in remote connectivity Proc AMIA Annu Fall Symp 1997 335 9 9357643 PMC2233594

[B37] Lorenzi N Riley RT Blyth AJC Southon G Dixon BJ Antecedents of the People and Organizational Aspects of Medical Informatics Review of the Literature J Am Med Inform Assoc 1997 4 2 79 93 9067874 10.1136/jamia.1997.0040079 PMC61497

[B38] Lorenzi NM Riley RT Organizational Aspects of Health Informatics: Managing Technological Change 1995 New York: Springer-Verlag

[B39] American Academy of Family Physicians AAFP pushes for affordable EMR system Family Practice Management Monitor 2003 http://www.aafp.org/fpm/20030200/monitor.html

[B40] eHealth Initiative Building Consensus for Common Action 2007 http://www.ehealthinitiative.org/blueprint/eHiBlueprint-BuildingConsensusForCommonAction.pdf

[B41] Veterans Health Information Systems and Technology Architecture (V*IST*A) http://www.va.gov/VISTA_MONOGRAPH/

[B42] WorldVistA 2008 http://worldvista.org/

[B43] California HealthCare Foundation Open-Source EHR Systems for Ambulatory Care: A Market Assessment 2008 http://www.chcf.org/topics/view.cfm?itemID=133551

[B44] California HealthCare Foundation Physician Practices Are Application Service Providers Right for You? 2006 http://www.chcf.org/topics/view.cfm?itemID=125716

[B45] Edsall RL Adler KG An EHR User-Satisfaction Survey: Advice from 408 Family Physicians Fam Pract Manag 2005 12 9 29 35 16296595

[B46] West Virginia e-Health Initiative So You Have Decided to buy an EHR... White paper 2005 http://www.wvehi.org/documents/WVeHIWhitePaper.pdf

[B47] Ryan S Choosing electronic health record systems Physicians News Digest 2006 http://www.physiciansnews.com/business/1006ryan.html

[B48] Medical Strategic Planning, Inc 2006 EHR Selector: Frequently Asked Questions for Physician, CIOs, EHR Consultants and EHR Developers https://www.ehrselector.com/ehrselector/EMRToolkit/Files/FAQ.pdf Revised January 9, 2008

[B49] Ash JS Bates DW Factors and Forces Affecting EHR System Adoption: Report of a 2004 ACMI Discussion J Am Med Inform Assoc 2005 12 1 8 12 15492027 10.1197/jamia.M1684 PMC543830

[B50] Alberta Health Services, Calgary Health Region Electronic Health Record Overview http://www.calgaryhealthregion.ca/cio/ci/projects/current/ehr/faq.htm

[B51] Zandieh SO Yoon-Flannery K Kuperman GJ Langsam DJ Hyman D Kaushal R Challenges to EHR implementation in electronic-versus paper-based office practices J Gen Intern Med 2008 23 6 755 61 Epub 2008 Mar 28 10.1007/s11606-008-0573-5 18369679 PMC2517887

[B52] Fullerton C Aponte P Hopkins R Bragg D Ballard DJ Lessons learned from pilot site implementation of an ambulatory electronic health record Proc (Bayl Univ Med Cent) 2006 19 4 303 10 17106488 10.1080/08998280.2006.11928188 PMC1618740

